# Personal protective equipment waste management behavior of undergraduates in Xi'an City based on extended theory of value-identity-personal norm model

**DOI:** 10.1038/s41598-023-36534-1

**Published:** 2023-07-10

**Authors:** Ting Li, Ting Xu, Yan Liang, Wei Luo, Jin Zhang

**Affiliations:** 1 Xi’an Eurasia University, Xi’an, China; 2 School of Architecture, Chang’an University, Xi’an, China; 3 Faculty of Built Environment and Surveying, Universiti Teknologi Malaysia, Johor, Malaysia; 4 Tsinghua University Architectural Design and Research Institute Co. LTD, Beijing, China; 5 Engineering Research Center of Urban Intelligent Construction, Universities of Shaanxi Province, Xi’an Eurasia University, Xi’an, China

**Keywords:** Environmental social sciences, Engineering

## Abstract

The COVID-19 pandemic has changed people’s lives, with the most prominent change being the use of Personal Protective Equipment (PPE). In this study, we used the extended Value-Identity-Personal (VIP) norm model to empirically analyze the influencing factors of Pro-Environmental Behavior (PEB) among college students in Xi 'an, China, while considering the usage of PPE as an example of PEB. We proposed nine hypothetical questions, and the VIP model was established through the SmartPLS software to test the valid questionnaires of 414 college students. The verification results indicated that all the nine hypotheses were supported statistically, with personal environmental social responsibility and personal norms showing the most significant direct impact on PEB; notably, personal norms were also strongly influenced by environmental personal social responsibility. Biosphere values affected PEB indirectly, through self-identity and individual norms. This study proposes viable countermeasures and suggestions for college students to improve PEB; our findings can serve as a reference for policymakers and stakeholders to ensure the effective waste management of personal safety equipment.

## Introduction

The COVID-19 pandemic caused a global health emergency that triggered an acute shortage of disposable protective equipment, including personal protective equipment (PPE). To date, the daily use of PPE equipment in China is still high, and the country faces a major concern regarding the safe disposal of PPE waste.

### Usage status of personal protective equipment (PPE) in China

Before 2020, the production of PPE in China increased gradually. According to the CCID data, the production of medical protective clothing in 2019 only increased by 9.18%, compared to that in 2018. In the same year, the total output of medical masks was 5 billion yuan, with an output value of 10.235 billion yuan. In 2020, the total output value of masks in China exceeded 70 billion yuan, an increase of nearly seven times that in 2019. In terms of the consumption of medical protective clothing, from January 2020 to April 2020, the disposable consumption of medical protective clothing in China was about 141 million pieces, about 33 times the annual consumption in 2019.

By 2021, due to the globalization and normalization of the epidemic, PPE, such as medical masks and protective clothing, were considered a protective necessity in people’s daily lives; therefore, the recycling and management of PPE remain a focus of waste management studies.

### Literature review of personal protective equipment (PPE) recycling and management

In general, PPE are disposable; studies have proven that PPE waste disposal during the epidemic had a significant negative impact on the environment^1–4^. Therefore, scholars conducted analysis from the perspectives of recycling and behavior management.

In terms of recycling from the viewpoint of chemistry (e.g., glycolysis, hydrogenation, aminolysis, hydrolysis, pyrolysis, and gasification), the components of contaminated PPEs should be meticulously managed and destructed through qualified waste accumulators, properly bagged, or reused to stop environmental pollution^5,6^. In terms of physical processes, for the decontamination of face coverings, PPE reuse can be achieved using a washing machine or spin dryer at 60 °C for 60 min, through supercritical CO_2_ sterilization, or other effective disinfection methods^7–9^.

From the perspective of behavior management, as PPE waste management behavior is considered as pro-environmental behavior (PEB), the theory of planned behavior (TPB) and its extended model have been widely used to predict pro-environmental behavior and behavior intention^10^. De Leeuw et al.^11^ applied the TPB model to analyze the eco-friendly behavior of high school students; other scholars focused on the behaviors of different groups, e.g., tourists, citizens, construction workers, and farmers^12–14^. Razali et al.^15^ added the moral norm into the TPB to determine the influencing factors of waste separation at the source in terms of the behavior of the residents in Malaysian households. Additionally, the studies also happen in the medical field, air pollution field, energy using field, etc^16^. The value-identity-personal (VIP) norm model is generally used to investigate the relationships between biospheric values, environmental self-identities, and personal norms in PEBs^17^.

In this study, we focused on PPE management behavior through the application of the VIP model to understand the psychological factors that influence the student population in the universities of China to perform PPE item management. Most studies on waste management and its influencing factors focus on urban residents and farmers, whereas only a few focus on the behaviors of college students. The level of waste recycling depends to a large extent on the effectiveness of the waste recycling policy (WRP)^18^. Therefore, a better understanding of the influencing factors that determine the behavior of younger generations could help authorities and stakeholders to develop stronger policies and increase the younger population’s willingness to participate in environmental policies and endeavors.

## Extended theory of value-identity-personal (VIP) norm model and hypotheses

### Theory of value-identity-personal (VIP) norm model

**Figure 1:**
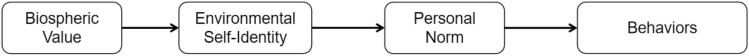
Value-identity-personal (VIP) norm model developed by Van der Werff and Steg (2016).

The VIP norm model was developed by Van der Werff and Steg^17^ (Fig. 1), with the aim of understanding the general determinants of environmental acts. The model consists of three components: biospheric values, personal norms, and environmental self-identity. The model postulates that environmental behaviors are affected by feelings related to the moral obligation to act in a setting (personal norms). Since the model was proposed recently, only a few studies have applied it to account for pro-environmental behavioral intentions and behaviors.

### Construction of extended theory of value-identity-personal (VIP) model and hypothesis analysis

**Figure 2:**
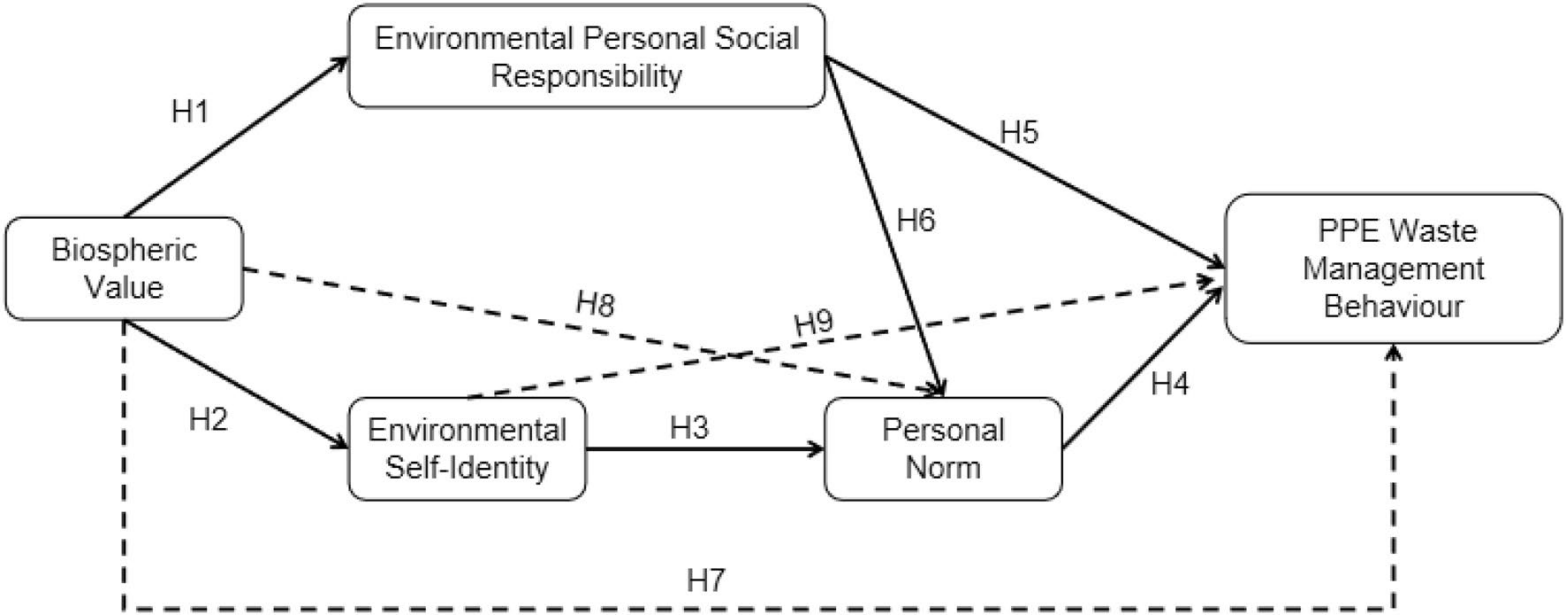
Conceptual framework proposed in this study; personal protective equipment (PPE).

Previous studies indicate that behaviors can be influenced by social responsibility^19,20^; however, there is limited knowledge regarding the influence of environmental personal social responsibility on PEB. Furthermore, studies should determine whether personal norms, defined as moral obligation, are affected by environmental personal social responsibility. In this article, we aim to merge environmental personal social responsibility into the VIP Model to explore the reasons why younger generations implement PEB. The study model is shown in Fig. 2.

#### Biospheric value

The construct of biosphere values was developed by Stern et al.^21^, who first proposed the concept of environmental values. They divided environmental values into three different value orientations: altruistic, egoistic, and biosphere values. Biospheric values consist of the main beliefs that emphasize thoughts about the biosphere and focus on the quality of environment, regardless of the usefulness it presents for people. Previous studies also reported that people who strongly validated biospheric values were concerned about the environment; they based their decision to take specific actions for the environment^22^. Biospheric values were associated with a wide range of pro-environmental preferences and behaviors^23,24^. In line with previous results, we present the following hypotheses:

Several studies indicate that environmental self-identity is influenced by biospheric values; stronger biospheric values result in a stronger environmental self-identity. Van der Werff et al.^24^ determined that the relationship between biospheric value and environmental preference, intention, and behavior was completely mediated by environmental self-identity. Though there is no literature on the relationship between environmental personal social responsibility and biosphere values, Ali and Mandurah^25^ observed that higher self-transcendence values led to higher levels of social responsibility among consumers. Therefore, we could hypothesize that:

##### H1

Biospheric value is positively related to environmental personal social responsibility.

##### H2

Biospheric value is positively related to environmental self-identity.

#### Environmental self-identity

According to Van der Werff et al.^24^, “acting environmentally friendly is an important part of who I am; I am the type of person who acts environmentally friendly; I see myself as an environmentally friendly person.” Chan et al.^26^ (2020) revealed that environmental self-identity was positively associated with personal norm (β = 0.04). On the basis of the reviewed literature, we proposed the following hypothesis:

##### H3

Environmental self-identity is positively related to personal norm.

#### Personal norm

Schwartz^27^ defined personal norms as “a kind of self-expectation that reflects the individual sense of responsibility for implementing specific actions.” Several scholars have confirmed that personal norms have a positive impact on individual behaviors. For example, Tina’s research has proved that personal norms are the main determinants of individual clothing consumption intentions and behavior. The study indicated that the higher the personal norms of youth, the better their PEB. Based on this perspective, we propose the following hypothesis:

##### H4

Personal norm is positively related to pro-environmental behaviors.

#### Environmental personal social responsibility

Protecting the environment is the responsibility of each inhabitant; we all have personal and social responsibilities towards the environment. In line with this perspective, Warne^19^ stated the ethical and social responsibilities of advertising and selling practices. Misener et al.^28^ indicated that the awareness of organizational social responsibility had a positive direct effect on the involvement behaviors of the members. Shah et al.^29^ conducted an analysis that was based on completed questionnaires and concluded that the perceived corporate social responsibility (CSR) activities generated the employees’ PEB.

Even though several studies suggest that social responsibility positively affects people’s behaviors and attitudes, only a few investigate the effects of environmental personal social responsibility (EPSR) on PEB. In general, having a sense of personal responsibility denotes being prepared to adhere to society’s norms of personal behavior. With respect to empirical evidence, we propose the following hypotheses:

##### H5

Environmental personal social responsibility is positively related to pro-environmental behaviors.

##### H6

Environmental personal social responsibility is positively related to personal norms.

#### Indirect relationship between constructs

In this study, we analyzed the indirect relationships between different constructs. We examined the indirect influence because, in a three-variable system, there are two causal paths that feed the outcome variable: the direct impact of the independent variable and the impact of the mediator. There is also a path from the independent variable to the mediator^30^. On this premise, we hypothesized the following:

##### H7

Biospheric value is positively related to pro-environmental behaviors.

##### H8

Biospheric value is positively related to personal norms.

##### H9

Environmental self-identity is positively related to PEBs.

## Methods

### Data collection and participants

In this study, we attempt to understand the factors that influence the PPE waste management behaviors of undergraduates in Xi'an, China. Due to the impact of the epidemic, we decided to distribute digital format questionnaires (Table 1) to undergraduates who is 18–24 years old in the urban area of Xi'an. The statistical method of this experiment data adopts random statistics. All subjects were randomly selected in the 3 universities, namely Xi'an Jiaotong University, Xidian University and Xi'an Eurasia University. We collected a total of 482 questionnaires, and then eliminated the invalid questionnaires like not being an undergraduate or incomplete questionnaire; finally, a total of 414 valid questionnaires were collected. The questionnaire was divided into two parts: A and B. Section A consisted of the basic information about the interviewees, such as age and gender. Through Pearson correlation analysis, we could deduce that PPE management behavior had a low correlation with the age and gender of the students (age |r|= 0.055, gender |r|= 0.064, both less than 0.5). Section B consisted of the main observation indicators, with a 5-point Likert scale that ranged from “Strongly Agree” to “Strongly Disagree”.

**Table 1:** Questionnaire used in this study.

Determinants	Indicators
Biospheric values	BV1: I feel an emotional connection with nature
	BV2: Humans can live in harmony with other species
	BV3: The environment are protected and preserved
	BV4: Nature and Ecosystem free from pollution
Environmental self-identity	EI1: Managing PPE waste in an environmentally-friendly manner is an important part of who I am;
	EI2: I see myself as a person who manages waste in an environment-friendly manner
	EI3: I am the type of person who manages PPE waste in an environmentally-friendly manner
Personal norm	PN1: I would feel guilty if I disposed of PPE in a way that was not environmentally-friendly
	PN2: I think I would be a better person if I were to act in an environmentally friendly way
	PN3: I feel a moral obligation to dispose of PPE waste in an environmentally friendly manner
Environmental personal social responsibility	EPSR1: I pay attention to environmental protections in daily life and consumption on PPE
	EPSR2: I make personal sacrifices to reduce PPE waste pollution
	EPSR3: I do not manage PPE waste in a manner that can potentially harm the environment
	EPSR4: I have stopped any PPE waste mismanagement action for environmental reasons
PPE waste management behavior	WMB1: I will stay home as much as possible to reduce the use of PPE
	WMB2: I will not dispose of PPE in an environmentally unfriendly manner
	WMB3: I can collect PPE separately from other garbage
	WMB4: I am able to dispose of PPE waste such as disposable masks, gloves, etc., properly

*PPE* Personal protective equipment.

### Data analysis

**Figure 3:**
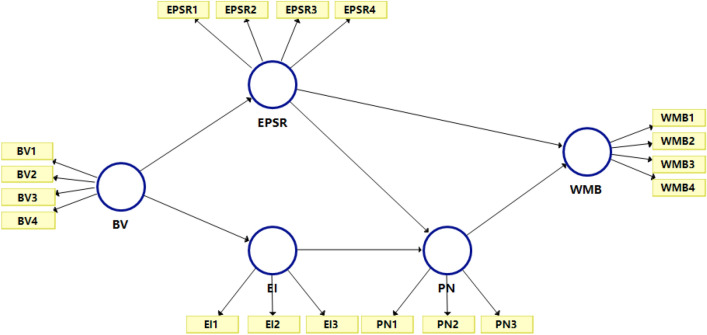
Partial least squares-structural equation modelling (PLS-SEM) framework portraying the influencing factors of personal protective equipment (PPE) waste management behaviors; the abbreviations are explained in Table 1.

Structural equation modeling (SEM) modeling for validity detection is mainly divided into covariance matrix method (CB) and partial least squares (PLS) estimation method. The PLS-SEM model is mainly applied for the exploratory analysis of the constructed model structure or the comprehensive analysis of the constructed composite index. In this study, by focusing on the influencing factors of the PPE waste management behaviors of undergraduate students in Xi’an (China) through exploratory and comprehensive analyses, we were able to deduce that the PLS-SEM model was suitable for such studies (Fig. 3).

In this study, we carried out data analysis, using Statistical Package for the Social Sciences (SPSS 22) modelling and SmartPLS 3. First, an exploratory factor analysis (EFA) was performed to identify the structure and factor loadings, using SPSS 22. Then, using SmartPLS 3, Cronbach’s Alpha, composite reliability (CR), and average variance extracted (AVE), we tested the validity and reliability of the proposed model. Furthermore, we used SEM to perform path analysis to examine the relationships among the constructs. Before performing EFA, we evaluated the multivariate normality and sampling adequacy of the model. As Bartlett’s test of sphericity yielded significant result (*p* < 0.05) and the value of Kaiser–Meyer–Olkin (0.874) was higher than 0.50^31^, we could conclude that the model was appropriate for the factorability of the data. During the EFA, we conducted principal component analysis with varimax rotation to extract the salient factors. The results of the EFA were acceptable, with the total variance explained being 72.16%^32^.

### Ethics declarations

The institutional review board of Xi’an Eurasia University approved the study protocol before data collection. Informed consent was obtained for all survey questionnaire participants. All methods were carried out in accordance with relevant guidelines and regulations.

## Results

### Partial least squares-structural equation modelling (PLS-SEM) model testing

To eliminate the dimensional difference of index data, the collected data must be standardized. In this study, we used the SmartPLS 3 software to construct and analyze the PLS-SEM model. To verify the accuracy of the SEM results, we checked the parameters, structure model, measurement model, and model fitting validity. The commonly used test methods mainly include reliability, validity, goodness of fit, and structural path significance tests.

#### Reliability testing

We used Cronbach’s Alpha and CR as measurement indexes; the measurement standards applied for this study are shown in Table 2.

**Table 2:** Cronbach’s Alpha and composite reliability (CR) standards.

Inspection Item	Testing value	Reference standard	Test criterion
Internal consistency reliability	Cronbach's Alpha	> 0.7 0.35 ≤ Cronbach’s Alpha < 0.7 < 0.35	High reliability Medium reliability Low reliability
Composite reliability	CR	> 0.7	Credible

To verify the rationality of the measured variables among the five latent variables in the index, we carried out a reliability test on the five latent variables; the test results are shown in Table 3. The values of Cronbach’s Alpha and CR in environmental personal social responsibility, environmental self-identity, personal norm, and PPE waste management behavior were all greater than 0.70 and thus, achieved the standard. The CR of biospheric values passed the test; however, Cronbach’s Alpha was 0.603 < 0.7, indicating that the values failed the test. Thus, the measurement indicators in the biospheric value need to be modified or deleted. By testing the correlation of measurement variables within each latent variable (Table 4), we could deduce that BV3 and BV4, which had a relatively low loading (below 0.7), need to be deleted; the test results are shown in Tables 5 and 6.

**Table 3:** Cronbach’s Alpha and composite reliability (CR) values for the five latent variables.

Latent variable	Cronbach’s Alpha	CR
Environmental personal social responsibility	0.878	0.916
Environmental self-identity	0.838	0.903
Personal norm	0.837	0.902
PPE waste management behavior	0.813	0.877
Biospheric value	0.603	0.77

*PPE* Personal protective equipment.

**Table 4:** Factor loadings of all the indicators considered in this study.

	Biospheric value	Environmental personal social responsibility	Environmental self-identity	Personal norm	PPE waste management behavior
BV1	0.809				
BV2	0.827				
BV3	0.644				
BV4	0.374				
EI1			0.891		
EI2			0.895		
EI3			0.819		
EPSR1		0.889			
EPSR2		0.819			
EPSR3		0.871			
EPSR4		0.84			
PN1				0.843	
PN2				0.869	
PN3				0.892	
WMB1					0.829
WMB2					0.852
WMB3					0.788
WMB4					0.728

*PPE* Personal protective equipment.

The abbreviations are explained in Table 1.

**Table 5:** Cronbach’s Alpha and composite reliability (CR) of the five latent variable after verification.

Latent variable	Cronbach’s Alpha	CR
Biospheric value	0.872	0.940
Environmental self-identity	0.838	0.903
Environmental personal social responsibility	0.878	0.916
Personal norm	0.837	0.902
PPE waste management behavior	0.813	0.877

*PPE* Personal protective equipment.

**Table 6:** Factor loadings of indicators after verification.

	Biospheric value	Environmental personal social responsibility	Environmental self-identity	Personal norm	PPE waste management behavior
BV1	0.938				
BV2	0.946				
EI1		0.892			
EI2		0.900			
EI3		0.813			
EPSR1			0.890		
EPSR2			0.818		
EPSR3			0.873		
EPSR4			0.838		
PN1				0.843	
PN2				0.868	
PN3				0.892	
WMB1					0.829
WMB2					0.852
WMB3					0.787
WMB4					0.728

*PPE* Personal protective equipment.

The abbreviations are explained in Table 1.

**Figure 4:**
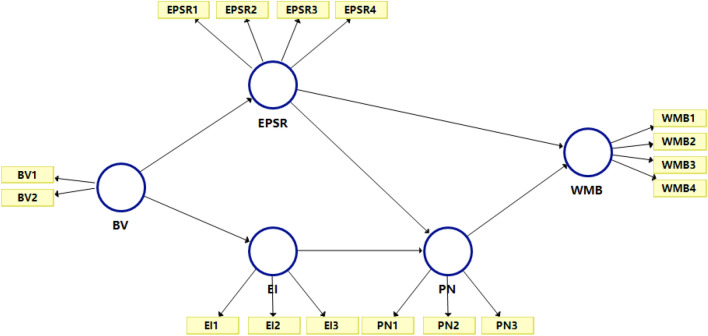
Modified partial least squares-structural equation modelling (PLS-SEM) model; the abbreviations are explained in Table 1.

After the PLS-SEM model was modified, the reliability and correlation tests of the model yielded good results, indicating that the selected data had good reliability. Therefore, in this study, we adopted the revised model for analysis. The PLS-SEM model of the influencing factors of PPE waste management behavior is shown in Fig. 4 below.

#### Validity testing

In general, validity test is used to analyze the correctness of the measured data, i.e., whether the measured variable can accurately measure the latent variable. Validity test mainly include discriminant and convergent validities, among which discriminant validity is used to detect the exclusivity problem, judged by whether the square root of the AVE value is greater than the correlation coefficient between the latent variables. Convergence validity is used to analyze the circumductility of latent variables. It is generally estimated using the AVE value, and its critical value is 0.5. The closer the AVE value is to 1, the better the validity test result. In this study, the discriminant and convergence validity tests of the five latent variables yielded good results, indicating that the constructed model had good structural validity (Tables 7, 8).

**Table 7:** Correlation between constructs.

	Biospheric Value	Environmental self-identity	Environmental personal social responsibility	Personal norm	PPE waste management behavior
Biospheric Value	**0.942**				
Environmental self-identity	0.474	**0.869**			
Environmental personal social responsibility	0.398	0.751	**0.855**		
Personal norm	0.451	0.752	0.762	**0.868**	
PPE waste management behavior	0.423	0.734	0.808	0.775	**0.801**

*PPE* Personal protective equipment.

The abbreviations are explained in Table 1.

Diagonal values show √AVE for each variable.

Significant values are in [bold].

**Table 8:** Average variance extracted (AVE) values for the five latent variables considered in this study.

	Biospheric value	Environmental self-identity	Environmental personal social responsibility	Personal norm	PPE waste management behavior
AVE	0.887	0.756	0.731	0.754	0.641

#### Goodness of fit

In this study, the goodness-of-fit of the PLS-SEM model was consistent with the regression analysis results, and the explanatory power of the model was verified by the coefficient of determination (R^2^). In general, R^2^ is an important basis for testing the quality of models and represents the explanatory ability of measured variables to latent variables. As shown in Table 9, in our study, the R^2^ value for the PPE waste management behavior endogenous latent variable was 0.714. This indicated that the three latent variables (EI, EPSR, PN) moderately explained 71.4% of the variance in the behaviors (Wong, 2019), with a pretty high goodness-of-fit value (R^2^ > 0.25). The predictive relevance (Q^2^) values were all above 0, which means the various have model’s predictive relevance.

**Table 9:** Coefficient of determination (R^2^) and predictive relevance (Q^2^) of endogenous various.

	Environmental self-identity	Environmental personal social responsibility	Personal Norm	PPE waste management behavior
R^2^	0.225	0.159	0.654	0.714
Q^2^	0.167	0.112	0.485	0.449

*PPE* Personal protective equipment

#### Structural path significance

In this study, we used bootstrapping algorithm to conduct 500 samplings of the PLS-SEM model; we obtained the test results of the model significance testing. We used a two-tailed t-test, with a significance level of 5%; the path coefficient was considered to be significant if the T-statistics were greater than 1.96^33^; the hypothesis was supported if P value is less than 0.05, as shown in Table 10, indicating that all of the structural model relationships were significant.

**Table 10:** Results of the proposed partial least squares-structural equation modelling (PLS-SEM) model.

Path	Path coefficients	T statistics	*P* value	Hypothesis	Hypothesis situation
Direct effect					
BV—>EPSR	0.398	7.087	***	H1	Supported
BV—>EI	0.474	10.371	***	H2	Supported
EI—>PN	0.411	6.856	***	H3	Supported
PN—>WMB	0.378	7.505	***	H4	Supported
EPSR—>WMB	0.520	10.760	***	H5	Supported
EPSR—>PN	0.453	8.030	***	H6	Supported
Indirect effect					
BV—>WMB	0.349	7.202	***	H7	Supported
BV—>PN	0.375	7.784	***	H8	Supported
EI—>WMB	0.155	4.743	***	H9	Supported

*, **, and *** Represent *P* values less than 0.1, 0.01–0.05, and less than 0.01, respectively.

The results of bootstrapping test of the PLS-SEM model portrayed the following:

1. Biospheric value was directly related to environmental personal social responsibility, thus, supporting Hypothesis H1
2. Biospheric value was directly related to environmental self-identity, thus, supporting Hypothesis H2
3. Environmental self-identity was directly related to personal norm, thus, supporting Hypothesis H3
4. Personal norm was directly related to PPE waste management behavior, thus, supporting Hypothesis H4
5. Environmental personal social responsibility was directly related to PPE waste management behavior, thus, supporting Hypothesis H5
6. Environmental personal social responsibility was directly related to personal norm, thus, supporting Hypothesis H6
7. Biospheric value was indirectly related to PPE waste management behavior, thus, supporting Hypothesis H7
8. Biospheric value was indirectly related to personal norms, thus, supporting Hypothesis H8
9. Environmental self-identity was indirectly related to PPE waste management behavior, thus, supporting Hypothesis H9

### Direct and indirect effects analysis

From the PLS-SEM algorithm, we determined the direct, indirect, and total effects, shown in Table 11.

**Table 11:** Effects of the proposed partial least squares-structural equation modelling (PLS-SEM) model.

Latent various	Path	Direct effects	Indirect effects	Total effects
BV	->EI	0.474		0.474
	->EPSR	0.398		0.398
	->PN		0.375	0.375
	->WMB		0.349	0.349
EI	->PN	0.411		0.411
	->WMB		0.155	0.155
EPSR	->PN	0.453		0.453
	->WMB	0.520	0.171	0.692
PN	->WMB	0.378		0.378

The abbreviations are explained in Table 1.

As shown in Table 11, environmental personal social responsibility and personal norms have a significant positive direct influence on PPE waste management behavior: environmental personal social responsibility (0.520) and personal norms (0.378).

In terms of the indirect effects, biospheric value indirectly affected the PPE waste management behavior and personal norms of the students through environmental self-identity and environmental personal social responsibility, with the total indirect effect coefficients being 0.349 and 0.375, respectively. Environmental self-identity indirectly affected the PPE waste management behavior of the students through personal norm, with an effect coefficient of 0.155.

From the perspective of total effect, among all latent variables, environmental personal social responsibility portrayed the greatest influence on the PPE waste management behavior, with a total effect coefficient of 0.692, followed by personal norm (0.378), biospheric value (0.349), and environmental self-identity (0.155).

### Analysis of empirical results

**Figure 5:**
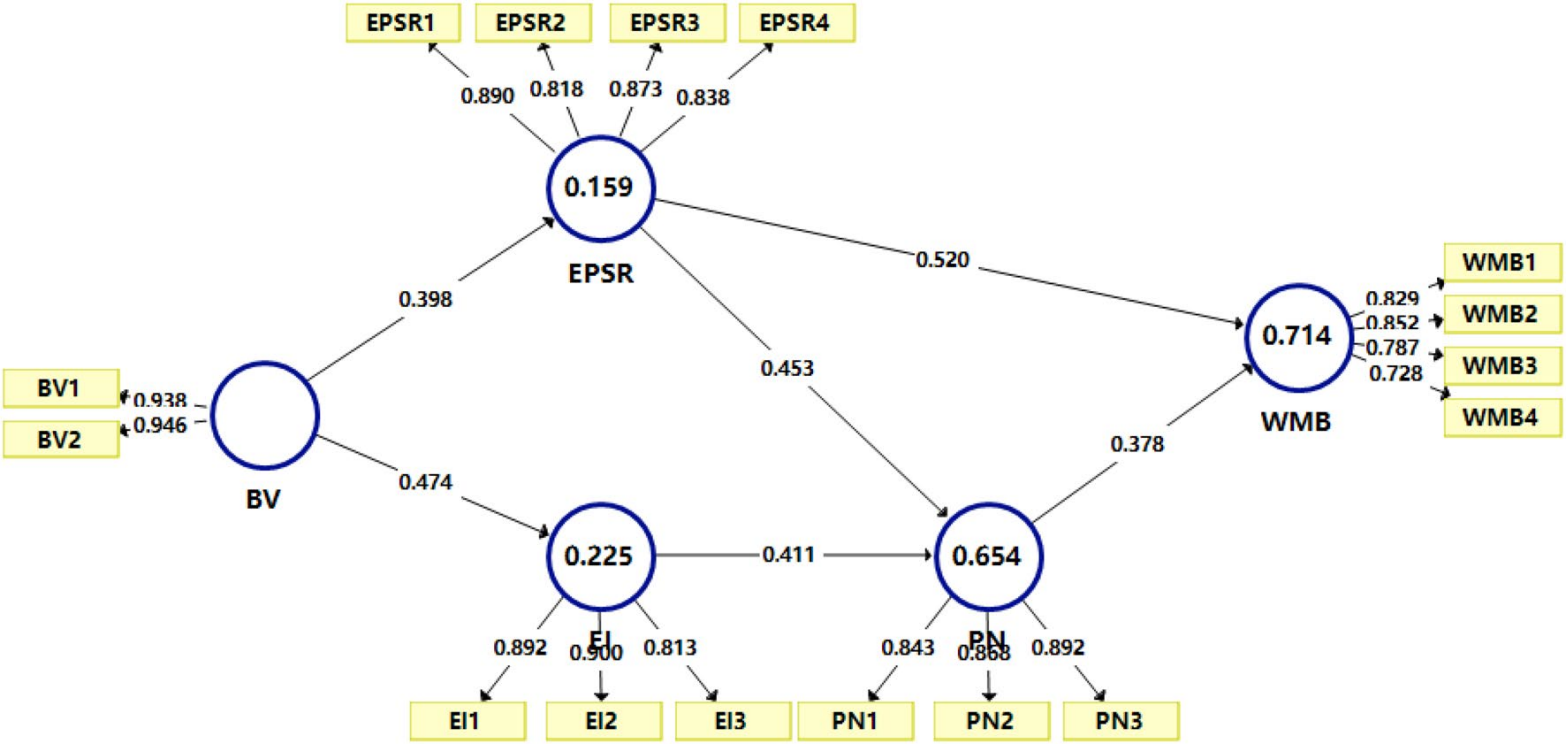
Final results of the partial least squares-structural equation modelling (PLS-SEM) algorithm applied in this study.

As shown in Fig. 5, through the empirical analysis of the influencing factors of PPE waste management behavior, we could determine the following five causal influence paths:

Path 1: Personal Norm—>PPE waste Management behavior;

Path 2: Biospheric Value—>Environmental self-identity—>Personal Norm—>PPE waste Management behavior;

Path 3: Environmental personal social responsibility—>PPE waste Management behavior;

Path 4: Biospheric Value—>Environmental personal social responsibility—>Personal Norm—>PPE waste Management behavior;

Path 5: Biospheric Value—>Environmental personal social responsibility—>PPE waste Management behavior.

Notably, all the latent variables portrayed a positive impact on PPE waste Management behavior.

## Discussion

The main purpose of this study was to determine the factors that influenced the PPE waste management behavior of the undergraduate students in Xi’an, China, within the scope of the extended VIP model. It is believed that young people are the main group of PPE consumption because of their high levels of activity; therefore, correct PPE management behavior among this group is crucial for the regional and global environment. In this study, we merged environmental personal social responsibility and the VIP model to obtain a robust conceptual model. The empirical results indicated that the model fitting results for the proposed model had an acceptable level. Notably, adding environmental personal social responsibility increased 11.4% of total variance of the proposed model, when compared to the basic VIP model (R^2^ = 0.60).

The findings confirmed that the VIP model explained the PEB of the students at the individual level in a parsimonious manner. First, the results indicated that biospheric values had a direct effect on the environmental self-identity, which implied that the stronger the biospheric value, the more strongly a person sees himself or herself as an environmental-friendly person and the more one is motivated to act in keeping with this self-identity. Additionally, we observed that environmental self-identity indirectly affected PPE waste management behavior via personal norm. This is consistent with the previous studies^24,34,35^. Moreover, environmental personal social responsibility was directly influenced by biospheric values; though there is no related previous study on this relationship, the finding is in line with the increasing empirical evidence that person responsibility is positively associated with values, such as biospheric values^36,37^.

Previous studies investigated the impact of personal social responsibility on environmental attitude^37^ or PEB and revealed the moderating effect between personal norms and behaviors^38^, but only a few cosndiered environmental personal social responsibility as a latent variable in the VIP model. In this study, the findings justified that environmental personal social responsibility had a significant and direct influence on PEBs and personal norm. Interestingly, the impact of environmental personal social responsibility on personal norm was stronger than that of environmental self-identity; additionally, the impact of environmental personal social responsibility on PPE waste management behavior was stronger than that of personal norm. This indicated that for college students, the level of personal environmental social responsibility was more likely to influence their PEBs; if a college student had a higher sense of social responsibility for the environment, they were more likely to choose an environment-friendly lifestyle. Thus, the results suggested the importance of environmental personal social responsibility in protecting the environment.

### Implications

Our study findings can have important implications for policymakers, universities, and governments and help promote the PEB in undergraduate students to ensure and encourage their pro-environmental lifestyle. Our study revealed that PPE waste management behaviors can be strongly determined by environmental personal social responsibility; personal norm was the secondary influencing factor and was strongly determined by environmental personal social responsibility. This implied that feelings of society obligation were major factors in determining the students’ intention and behaviors. The stronger a person’s environmental personal social responsibility, the stronger a person’s personal norm and the stronger a person’s will to engage in PEB.

In terms of the importance of environmental issues, it is crucial for universities and governments to prepare students to become active citizens, who can promote and develop a sustainable world, while Xi’an is a place with a concentration of higher education resources, producing over 300,000 undergraduate students from all over the country yearly. Accordingly, enhancing students’ environmental personal social responsibility may be the most rapid and efficient way to achieve domestic sustainability.

To achieve this goal, policy makers should strengthen the improvement of relevant laws and regulations, so that government administrators can punish individuals or groups who violate social responsibilities and obligations according to the law, and urge undergraduate students to be vigilant against behaviors that do not fulfill social responsibilities. Xie et al.^39^ found that incorporating historical materials related to chemistry can effectively enhance high school students' sense of chemical safety and social responsibility. Thus, for universities, it is essential to help students fully understand and recognize the relationship between the environmental awareness, actions and the health and safety of the ecological environment, and this could be achieved by setting some compulsory course or extracurricular activities.

### Limitation and scope for future studies

There are limitations in the study that should be given more attention in the future. First, the sample method was limited to convenience sampling and several hypotheses; future studies should involve larger sample data and focus on determining a more representative sampling method, using better tools. Next, this study only added the “environmental personal social responsibility” element as the extended determinant in the VIP model, even though many other variables could be included; in future studies, some of the potential variables could be the TPB, past experience, and environment knowledge. Moreover, although the model proposed in this study has certain universality, it was limited to the undergraduate students in Xi’an; therefore, the results may not be indicative of other groups or regions. When used in other groups (the elderly or children) or regions, the results may varies based on different data set. All of these limitations must be considered as extended determinants in future studies to promote further detailed studies and more extensive analyses of PPE waste management behaviors in not just China, but also other countries.

## Conclusion

The study results indicated that the undergraduates’ PPE waste management behaviors could be extended to the VIP model effectively. The determinants considered in this study were biospheric value, environmental self-identity, personal norm, and environmental personal social responsibility, all of which were significant and positively influenced the PPE waste management behaviors. The results of the 414 total questionnaires validated the role of the determinants in guiding the PPE waste management behaviors. Remarkably, the study proved that environmental personal social responsibility was the most influential determinant of PEBs. And environmental personal social responsibility had a positively influencing effects on personal norm. In spite of the scarcity of the existing studies’ attempts to merge environmental personal social responsibility into the VIP model, we were able to present specific and meaningful conclusions and express some degree of value and original view on this issue.

The study demonstrated the importance of environmental and personal social responsibility in promoting pro-environmental behaviours among the undergraduates, which has implications for sustainability efforts at both local and global levels. We recommend that environmental personal social responsibility be added to the education map of college students. Additionally, visits and field trips to rivers, green belts, and polluted areas should be organized and guided to deepen the understanding of environmental protection and enhance the students’ personal social responsibility and will for environmental protection.

## Data Availability

The datasets used and/or analysed during the current study are available from the corresponding author on reasonable request.

## Abbreviations

PPE: Personal protective equipment
PEB: Pro-environmental behavior
VIP: Value-identity-personal
WRP: Waste recycling policy
CSR: Corporate social responsibility
EPSR: Environmental personal social responsibility
SEM: Structural equation modeling
CB: Covariance matrix method
PLS: Partial least squares estimation method
SPSS: Statistical package for the social sciences
EFA: Exploratory factor analysis
CR: Composite reliability
AVE: Average variance extracted
PLS-SEM: Partial least squares-structural equation modelling
R^2^: Coefficient of determination
